# Machine learning identifies proteomic risk factors across 23 diseases

**DOI:** 10.1016/j.isci.2026.114687

**Published:** 2026-01-14

**Authors:** Lingqi Meng, Mengzhen Li, Xiangtai Kong, Tonghua Zhang, María Bueno Álvez, Xinmeng Liao, Hasan Türkez, Ozlem Altay, Cheng Zhang, Mathias Uhlén, Adil Mardinoglu

**Affiliations:** 1 Science for Life Laboratory, KTH Royal Institute of Technology, 17165 Stockholm, Sweden; 2 Department of Medical Biology, Faculty of Medicine, Atatürk University, Erzurum, Turkey; 3 Centre for Host-Microbiome Interactions, Faculty of Dentistry, Oral & Craniofacial Sciences, King’s College London, London SE1 9RT, UK

**Keywords:** proteomics, machine learning, medicine

## Abstract

Achieving minimally invasive and rapid detection is a crucial goal in modern medicine. The comprehensive characterization of the blood proteome holds great promise in advancing our understanding of disease etiology, facilitating early diagnosis, risk stratification, and improved monitoring across various diseases and their subtypes. In this study, we collected plasma proteomes from over 3000 patients, representing 23 distinct diseases, encompassing a total of 1462 proteins. Based on histological knowledge, we developed a two-stage hierarchical multi-disease classifier and applied it to perform multi-disease classification on the collected proteomic data. Our results demonstrate that this empirically guided two-stage hierarchical multi-disease classifier outperforms traditional machine learning algorithms in terms of prediction performance, showing better balance and more meaningful feature selections. This finding highlights the positive role that domain expertise can play in machine learning-based disease detection, and underscores the potential of plasma proteomics for multi-disease screening.

## Highlights

• Plasma proteomics enables multi-disease detection across 23 conditions
• A knowledge-based hierarchical classifier outperforms standard ML models
• Key proteins (e.g., FCGR3B, ITGB1BP1) show broad predictive importance
• Model achieves strong disease-vs-healthy discrimination (AUROC often >0.9)

## Graphical abstract

**Figure:**
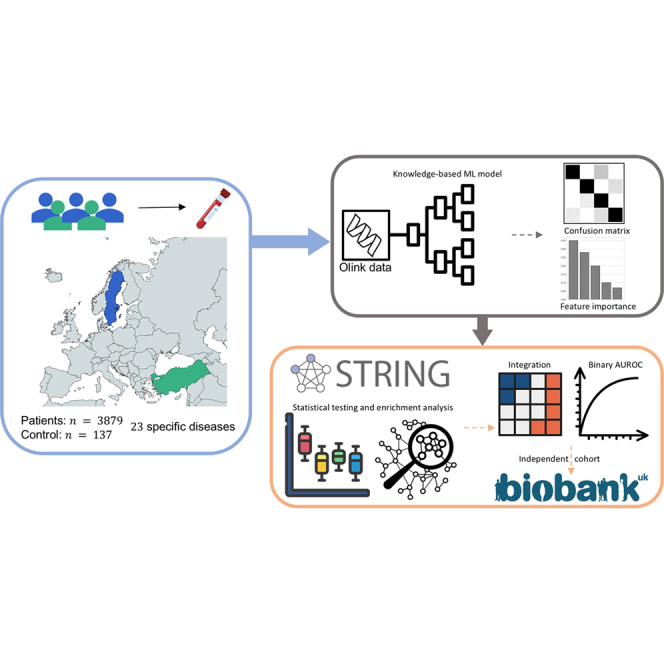

## Introduction

Proteins play an indispensable role in most biological processes, particularly in immune responses.^1^^,^^2^^,^^3^ Blood, as a crucial component of the circulatory system, carries a wide array of inflammatory proteins associated with immunity. By analyzing the specific spectra of individual inflammatory proteins, a comprehensive understanding of an individual’s disease condition can be obtained, including processes that may lead to carcinogenesis, such as abnormal tissue growth and proliferation.^4^^,^^5^^,^^6^ The abundance of proteins in the blood can largely be explained by genetic variations, as proteins are integral components of the circulatory system. However, this association is not always deterministic, as confounding factors and other epidemiological biases can influence the results.^7^

In contrast to the emphasis in traditional precision medicine on genomics and transcriptomics, proteins directly participate in phenotypic expression and biological processes, such as structural, enzymatic, and defense functions.^8^^,^^9^ Particularly, the overall composition of plasma inflammatory proteins reflects an individual’s immune processes. In the context of precision medicine, analyzing proteins from minute blood samples for liquid biopsy analysis presents an intriguing approach.^4^^,^^10^^,^^11^ However, the dynamic range of protein concentrations in blood is vast, spanning at least ten orders of magnitude, making multi-analyte analysis challenging even for a limited number of protein targets.^12^

In recent years, with the development of high-throughput platforms for sensitive protein profiling in blood, such as Somascan and the Proximity Extension Assay (PEA), the landscape has shifted. These platforms enable the simultaneous analysis of thousands of target proteins using only a few microliters of blood sample, and can detect and quantify proteins at levels as low as femtomoles.^4^^,^^6^^,^^13^ This advancement implies that even proteins present at concentrations far below the detection threshold of mass spectrometry can now be accurately detected and used for population screening.

However, to date, only certain cancers (such as breast cancer, cervical cancer, colorectal cancer, and lung cancer) meet the screening guidelines recommended by the US Preventive Services Task Force.^14^ While the use of these single cancer screening tests has reduced cancer-related mortality for these malignancies, early screening using single tests is evidently insufficient for individuals at high risk of multiple diseases. Many chronic inflammations (e.g., chronic hepatitis) are key contributors to carcinogenesis, placing a heavy financial burden on national health systems every year.^11^^,^^15^

In recent years, digital medicine, as an interdisciplinary field combining information science and medicine, has aimed to achieve rapid, high-resolution personalized diagnosis using high-dimensional data such as omics data. It can identify disease subtypes and provide optimized treatment and monitoring plans for individuals. Through digital medicine, inflammatory diseases and cancer can be detected early, initiating treatment to improve prognosis and preventing tumor progression, metastasis, and the emergence of refractory tumors.^4^^,^^11^^,^^13^^,^^16^^,^^17^ To date, several machine learning-based early detection tools for diseases have emerged, such as Galleri, a targeted methylation test based on circulating free DNA (cfDNA), which can detect a common signal across more than 50 cancer types,^18^ and MILTON, which uses genomic and proteomic data with phenotype associations for early detection.^19^ However, the FDA has yet to approve the commercial application of any multi-disease machine learning screening method.

Machine learning, a branch of computer science that has seen widespread application in the past decade, has become increasingly prevalent across various fields of biomedicine.^17^^,^^20^^,^^21^ However, because proteomic sequencing is still in its developmental phase, the available sample data are often characterized by small sample sizes and high dimensionality. This presents significant challenges for direct deep learning training on such data.^22^ To date, there has been no reliable pre-trained model for multi-disease proteomics based on PEA technology. Traditional machine learning models aim to identify appropriate decision boundaries in multi-class sample spaces and perform well in classification tasks with small sample sizes. However, classical models, which rely on predefined algorithms, often lack interpretability in the context of biomedical problems.^23^

To address these challenges, we propose a knowledge-based hierarchical classifier, a classification model constructed through the serial and parallel combination of multiple classical machine learning models. We apply a “divide and conquer” approach, initially categorizing the raw data into broad modules such as blood disorders, psychiatric diseases, metabolic disorders, and cancer. Each module is then further subdivided for more detailed classification. To tackle the issue of class imbalance, we applied an interpolation-extrapolation method for oversampling in each classifier.^24^

Our results demonstrate that this empirically guided two-stage hierarchical multi-disease classifier outperforms traditional machine learning algorithms in terms of prediction performance, including accuracy, weighted F1 score, and macro F1 score, showing better balance and more meaningful feature combinations. Furthermore, we conducted an enrichment analysis on the top 100 features selected by the algorithm, revealing that it provides a more comprehensive enrichment of pathway-related information compared to conventional algorithms. We have summarized these findings in a protein-protein interaction network.

## Results

### Multi-disease cohort

**Figure 1:**
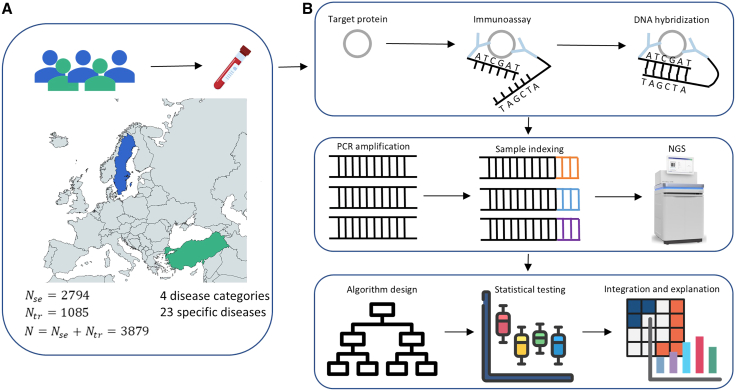
Cohort demographics and Olink proteomics workflow (A) Sample sources and demographic characteristics of the cohort. (B) Workflow of Olink technology and the analysis pipeline based on Olink proteomics data.

**Figure 2:**
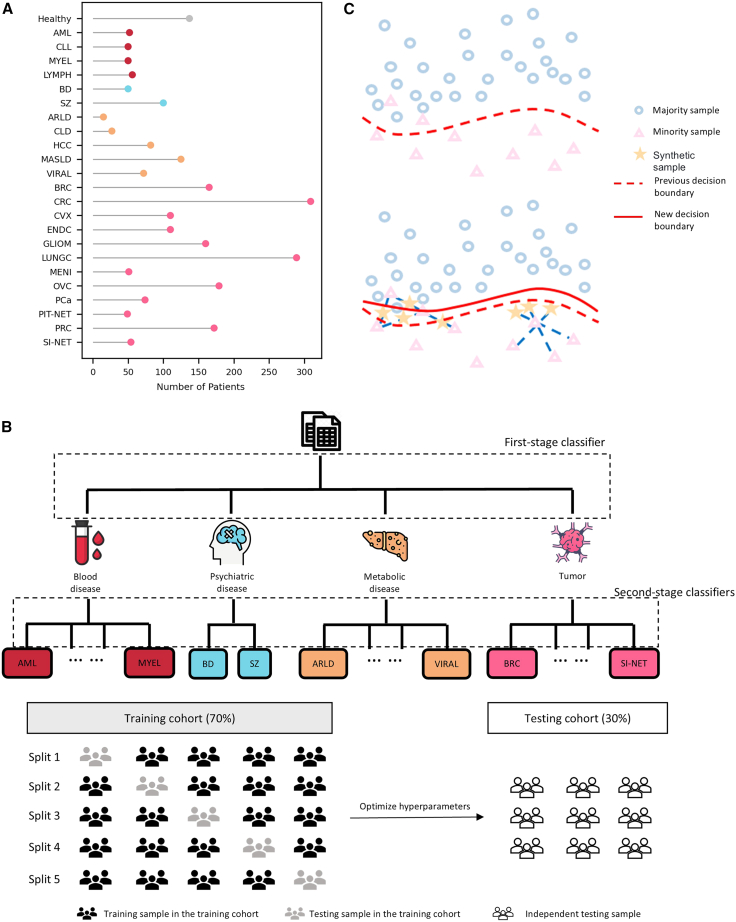
Sample distribution and model training framework (A) Distribution characteristics of the samples. Red represents blood diseases, blue represents psychiatric diseases, yellow represents metabolic diseases, and pink represents tumors. (B) Workflow and training process of the knowledge-based two-stage hierarchical model. (C) Workflow of borderline-SMOTE. Blue circles represent majority class samples, pink triangles represent minority class samples, yellow stars represent synthetic samples, and the new decision boundary is indicated by a solid red line.

In this study, we characterized the plasma proteome of a multi-disease cohort derived from two biobanks: the Uppsala-Umeå Comprehensive Cancer Consortium (*n* = 2,794) and the Anatolian Precision Medicine Initiative (*n* = 1,085). The combined cohort comprised 3,879 patients spanning four major disease categories: blood diseases, psychiatric diseases, metabolic disorders, and tumors (Figure 1A; the four major disease categories are highlighted in Figure 2B). Additionally, the cohort included a healthy control group (*n* = 137) and 23 specific disease groups, such as acute myeloid leukemia (AML; *n* = 52), chronic lymphocytic leukemia (CLL; *n* = 50), diffuse large B-cell lymphoma (LYMPH; *n* = 56), myeloma (MYEL; *n* = 50), bipolar disorder (BD; *n* = 50), schizophrenia (SZ; *n* = 100), alcohol-related liver disease (ARLD; *n* = 15), chronic liver disease (CLD; *n* = 27), hepatocellular carcinoma (HCC; *n* = 82), metabolic dysfunction-associated steatotic liver disease (MASLD; *n* = 125), viral hepatitis (VIRAL; *n* = 72), breast cancer (BRC; *n* = 165), colorectal cancer (CRC; *n* = 309), cervical cancer (CVX; *n* = 110), endometrial cancer (ENDC; *n* = 110), glioma (GLIOM; *n* = 160), lung cancer (LUNGC; *n* = 289), meningioma (MENI; *n* = 51), ovarian cancer (OVC; *n* = 179), pancreatic cancer (PCa; *n* = 74), pituitary neuroendocrine tumor (PIT-NET; *n* = 49), prostate cancer (PRC; *n* = 172), and small intestine neuroendocrine tumor (SI-NET; *n* = 54) (Table S1 and Figure 2A).

Plasma samples were collected at the time of diagnosis and prior to the initiation of any treatment. Detailed summary statistics for the disease cohorts, including age, sex, BMI, and stage distribution, are provided in Table S1. The sex distribution is also summarized in Figure S1.

### Olink technique and analysis workflow

To quantify the relative concentrations of immune proteins, we employed the Olink technique, a state-of-the-art high-throughput proteomics platform that leverages PEA technology for the precise and simultaneous quantification of multiple proteins. The method relies on the use of two antibodies, each conjugated to a unique DNA oligonucleotide, which bind to distinct epitopes on the target protein. Upon antibody binding, the DNA tags are brought into close proximity, enabling a DNA extension reaction that generates a unique barcode sequence. This sequence is subsequently amplified via polymerase chain reaction, and the resulting signal intensity is directly proportional to the concentration of the target protein (Figure 1B). Figure 1B provides a comprehensive overview of the workflow we implemented to identify multi-disease classification models, integrating the Olink-derived protein data with advanced statistical methods for robust analysis.

### Knowledge-based hierarchical classifier design

We built a two-stage hierarchical classification model used to predict diseases, leveraging both first and second stage classifiers (Figure 2B). The first stage classifier distinguishes among four main disease categories: blood disease, psychiatric disease, metabolic disease, and tumor. Within each category, second-stage classifiers further specify the disease type, such as AML, CLL, LYMPH, and MYEL for blood diseases; BD and SZ for psychiatric diseases; ARLD, CLD, HCC, MASLD, and VIRAL for metabolic diseases; and BRC and other tumor types in the tumor category. The “knowledge-based” design is reflected in clinical taxonomy guidance: the four disease categories (blood/psychiatric/metabolic/tumor) reflect established clinical classifications endorsed by the World Health Organization International Classification of Diseases, ensuring biological coherence. The model was trained using a 70% training cohort, using 5-fold cross-validation. The remaining 30% of data were used for testing. The results from the testing cohort were used to evaluate the model’s performance, providing an independent testing sample to confirm the accuracy and reliability of the predictions.

To improve the model’s performance by alleviating the impact of class imbalance, we apply the borderline-SMOTE (Synthetic Minority Over-sampling Technique) algorithm (Figure 2C). In the upper panel, the original decision boundary (represented by the dashed red line) is shown with the majority samples (blue circles) and minority samples (pink triangles) distributed across the feature space. The decision boundary, based on the original dataset, fails to properly classify the minority samples, as they are scattered in a region dominated by majority samples. In the lower panel, the borderline-SMOTE algorithm generates synthetic samples (depicted as yellow stars) near the decision boundary to enhance the minority class representation. These synthetic samples are strategically placed in regions where minority samples are misclassified, thereby improving the decision boundary (shown as the solid red line) to better separate the minority class from the majority class. The new decision boundary is adjusted to incorporate these synthetic samples, resulting in a more balanced and effective classification model.

### Prediction performance

**Figure 3:**
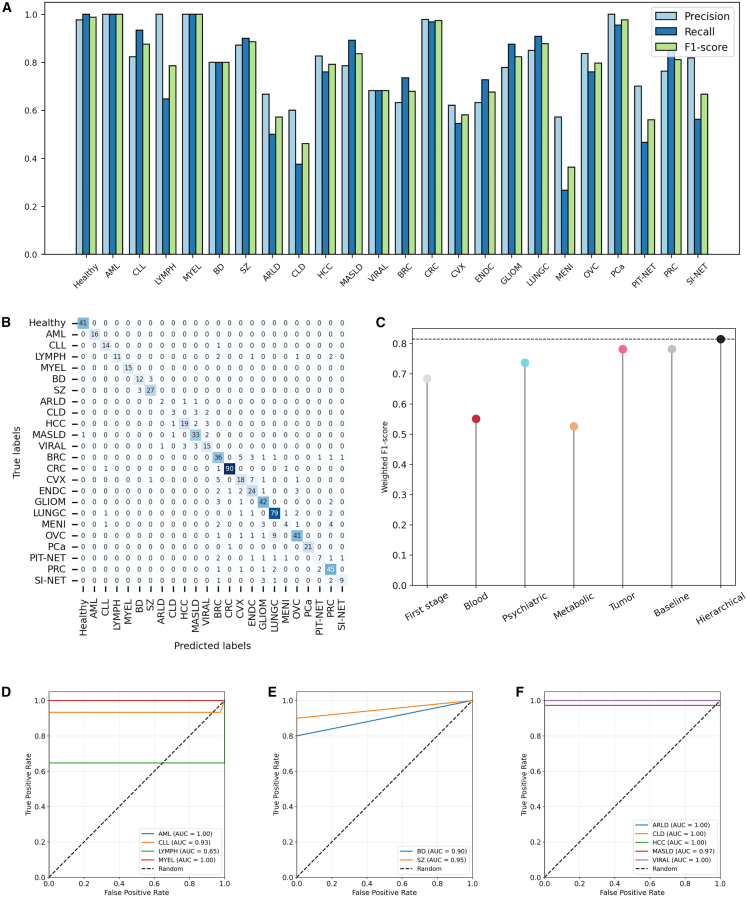
Performance evaluation of the two-stage hierarchical classification model (A) Classification performance of the two-stage hierarchical model, evaluated using precision, recall, and F1 score. (B) Confusion matrix of the two-stage hierarchical model. (C) Classification performance of each sub-model and the baseline model. (D–F) AUROC of the two-stage hierarchical model restricted to binary classification of specific diseases versus healthy controls: (D) blood diseases, (E) psychiatric diseases, and (F) metabolic diseases.

We showed the performance metrics of our machine learning model, including precision, recall, and F1 score (Table S2 and Figure 3A). The model demonstrates consistent performance overall, with precision, recall, and F1 score values predominantly ranging between 0.6 and 1.0. As expected, the model exhibits high discriminative capability in classifying hematological diseases, achieving 1.0 accuracy for AML and MYEL. However, its performance in classifying LYMPH subtypes is suboptimal, with a recall of 0.65 and an F1 score of approximately 0.79. For psychiatric disorders, the model shows reliable performance in classifying BD and SZ, with F1 scores of 0.80 and 0.89, respectively. In the case of metabolic diseases, the model’s performance varies, demonstrating poor classification accuracy for ARLD and CLD but achieving moderate performance for the other three metabolic conditions. Regarding cancer classification, the model achieves near-perfect performance (F1 score >0.97) for CRC and PCa, with F1 scores of approximately 0.85 for GLIOM, LUNGC, and PRC. However, its performance is less robust for other cancer types, particularly those associated with female-specific cancers, where the F1 score ranges from 0.58 to 0.8.

The confusion matrix illustrated in Figure 3B (Table S3) offers a comprehensive overview of the classification performance of our machine learning model across various disease categories. The matrix underscores the model’s proficiency in accurately predicting true labels, as evidenced by the substantial number of instances correctly classified along the diagonal. Nonetheless, certain misclassifications are evident, particularly among diseases within the same category, which may be attributed to overlapping features or inherent similarities in the data. Specifically, within the realm of psychiatric disorders, there is potential for confusion between BD and SZ (*n* = 6). In the context of metabolic diseases, instances of misclassification are observed among ARLD, CLD, and VIRAL (*n* = 13). Additionally, certain female-related cancers, such as CVX, ENDC, and OVC, may be erroneously classified as BRC (*n* = 8). The confusion matrix on the training cohort can be found in Figure S2A. We deconstructed the two-stage hierarchical model into its individual components and independently evaluated their predictive performance on the dataset. The results were then compared with the best-performing multi-class baseline model (Figure 3C). As clearly demonstrated, the classification performance of these individual component classifiers varied, with scores ranging from 0.53 to 0.78. However, when combined as a two-stage hierarchical model, these components achieved a significantly higher level of performance, yielding a weighted F1 score of 0.814.

To assess whether demographic variables influenced model performance, we performed post hoc stratification analyses based on sex, age, and BMI. To evaluate sex-related bias, we excluded sex-specific phenotypes (BRC, CVX, ENDC, PRC, and OVC) and assessed model performance separately in male-only and female-only test subsets across the remaining 19 phenotypes. The Mann-Whitney U test (significance level = 0.01) revealed no significant differences between the two groups (precision *p* = 0.94; recall *p* = 0.71; F1 score *p* = 0.58). Similarly, stratification by age (≥60 vs. <60 years) and BMI (>25 vs. ≤25) yielded no significant differences in model performance (age: precision *p* = 0.74, recall *p* = 0.49, F1 score *p* = 0.49; BMI: precision *p* = 0.84, recall *p* = 0.53, F1 score *p* = 0.72). These results suggest that the hierarchical model’s predictive capability is not systematically affected by sex, age, or BMI. Given that Olink NPX values capture disease-associated molecular profiles that may inherently reflect some demographic effects, the model appears to generalize well across population subgroups.

We further investigated the performance of the multi-class classification model in a binary classification setting, specifically focusing on subsets of the dataset comprising only the disease cohort of interest and the healthy control cohort. For example, for binary classification analyses (e.g., healthy vs. breast cancer), probabilities for the two relevant classes were extracted from the 24-dimensional multiclass output vector of the trained model and renormalized to sum to one. No additional model retraining was performed for these analyses. The predictive capability of the model under this binary classification constraint was evaluated using the Area Under the Receiver Operating Characteristic (AUROC) curve. The results demonstrate that, with the exception of LYMPH, the model exhibits strong performance in distinguishing hematological diseases from healthy controls, achieving AUROC scores greater than 0.93 (Figure 3D). Similarly, in the differentiation of psychiatric disorders from healthy individuals, the model also achieves AUROC scores exceeding 0.9 (Figure 3E). Moreover, it is noteworthy that, despite the observed limitations in the intra-category classification of metabolic diseases and tumors as illustrated in Figure 3A, the model still demonstrates a remarkable ability to distinguish these disease categories from the healthy cohort (Figures 3F, S2B, and S2C).

### Algorithm comparison

**Figure 4:**
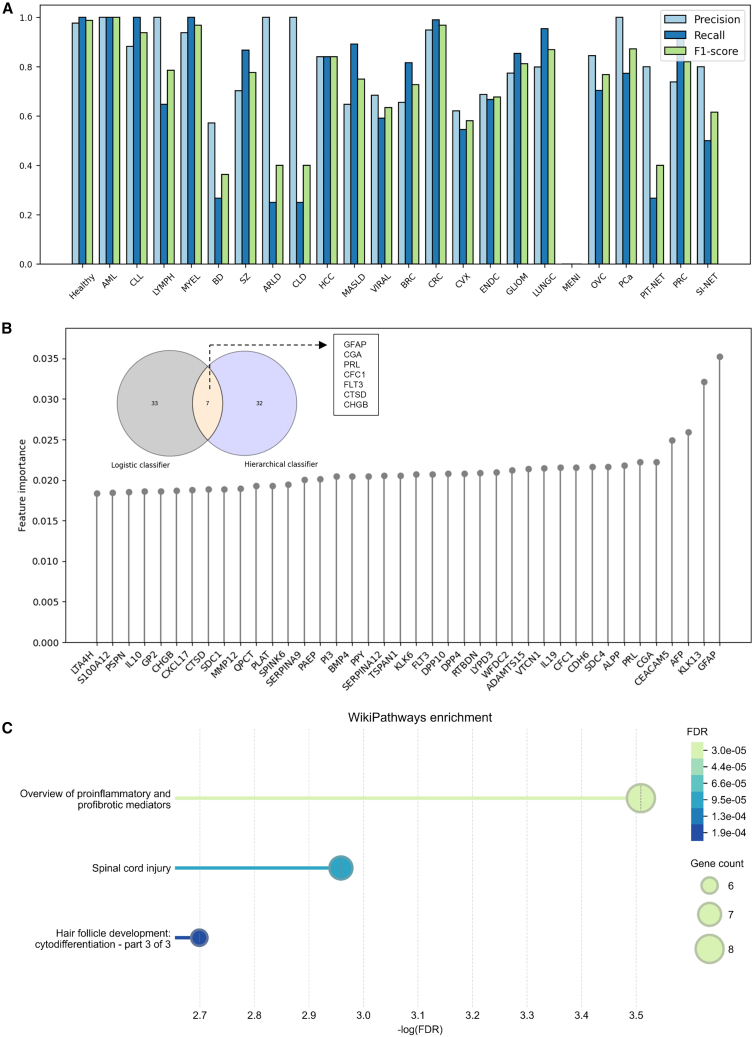
Feature importance and pathway analysis based on logistic regression (A) Classification performance of logistic regression. (B) Top 40 important features from the logistic regressor and their overlap with features derived from the two-stage hierarchical model. (C) WikiPathways enrichment analysis for the top 100 proteins from the logistic regressor.

We evaluated the performance of the baseline model (multiclass logistic regression) in predicting multiple disease categories. Similar to Figure 3A, we examined its performance metrics, including precision, recall, and F1 score (Table S4 and Figure 4A). Overall, we found that our hierarchical model outperformed the baseline model, achieving a 2.0% higher accuracy (a relative improvement of 2.5%), a 3.3% higher weighted F1 score (a relative improvement of 4.2%), and a 6.3% higher macro F1 score (a relative improvement of 8.9%; Table S4). It is evident that the baseline model demonstrated excellent performance (F1 score >0.95) in the healthy cohort, as well as in certain blood diseases such as AML and CLL, and certain cancers such as CRC. However, the model’s performance varied significantly across different diseases. For instance, the F1 score for BD was only 0.36, and for MENI, it was 0. This discrepancy may be attributed to the lack of oversampling for the minor classes. To address this, we further investigated the performance of the baseline model after training on an oversampled dataset. We found that borderline-SMOTE significantly improved the model’s classification capability for MENI, increasing the F1 score from 0 to 0.45. For BD, the F1 score improved from 0.36 to 0.65, although it remained lower than the 0.8 achieved by our two-stage hierarchical model (Table S5). We extracted the top 40 features from both the two-stage hierarchical model and the baseline model. Although the predictive performance of the two models was similar, the features they prioritized differed significantly. Among the top 40 features, only seven were shared between the two models (Figure 4B). In the baseline model, the most influential feature was the protein glial fibrillary acidic protein (GFAP), which is the primary intermediate filament protein in mature astrocytes and also plays a critical role in the cytoskeleton of astrocytes during development. This was followed by the proteins kallikrein-related peptidase 13 (KLK13), alpha-fetoprotein (AFP), and CEA cell adhesion molecule 5 (CEACAM5), all of which are well-known target genes in cancer research (Table S6 and Figure 4B).

Additionally, we performed a WikiPathways enrichment analysis on the top 100 important proteins. The results revealed that these proteins were predominantly enriched in the following pathways: 1. proinflammatory and profibrotic mediators (FDR <4 × 10−5), 2. spinal cord injury (FDR <10−4), and 3. hair follicle development: cytodifferentiation (FDR <2 × 10−4). The gene counts for the enriched pathways ranged between 6 and 8, indicating a moderate yet focused involvement of key genes in these biological processes (Figure 4C).

### Protein importance ranking and statistical significance

**Figure 5:**
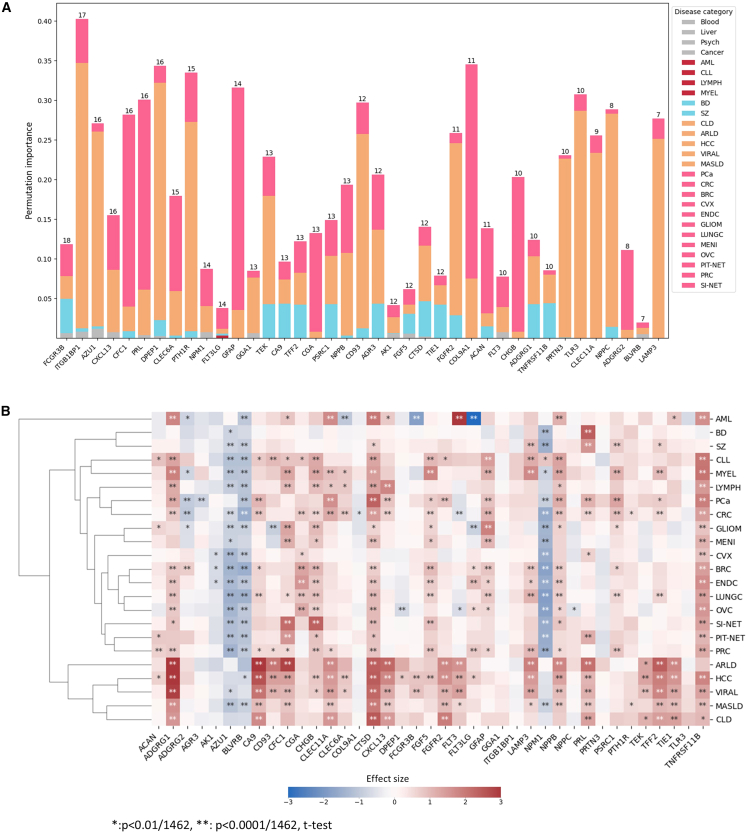
Disease-associated protein importance identified by the two-stage hierarchical model (A) Top 40 stacked feature importance from the two-stage hierarchical model, ranked by the occurrence of positive prediction contributions across diseases. (B) Statistical significance and effect size of the top 40 important proteins compared to the healthy cohort, clustered by disease. A *t* test was used, where ∗ represents *p* < 0.01/1,462, and ∗∗ represents *p* < 0.0001/1,462.

We evaluated the importance of each protein in the two-stage hierarchical model from two perspectives: (i) the frequency of each protein’s appearance in specific classifications, and (ii) the score assigned to each protein by the classifier. Intuitively, a protein that appears more frequently in (i) may play a significant role across multiple diseases, while a protein with a higher score in (ii) indicates its critical contribution to the model’s inference. In our importance analysis, the top three proteins based on frequency of appearance were Fc gamma receptor IIIb (FCGR3B), integrin subunit beta 1 binding protein 1 (ITGB1BP1), and dipeptidase 1 (DPEP1) (Table S7 and Figure 5A). It has been reported that FCGR3B is significantly downregulated in AML,^4^^,^^25^ and its copy number variation is associated with susceptibility to systemic autoimmunity.^26^ We observed that ITGB1BP1 plays a crucial role in the classification of liver diseases, with a score exceeding 0.3. Previous studies have shown that PAK proteins and YAP-1 signaling downstream of integrin beta-1 in myofibroblasts promote liver fibrosis.^27^ DPEP1, on the other hand, is a well-known biomarker that inhibits tumor cell invasiveness and enhances chemosensitivity in CRC and PCa.^28^^,^^29^ Using a *t* test, we found that each protein among the top 40 important proteins identified by our algorithm was statistically significant (*p* < 0.01/1462 or *p* < 0.0001/1462) in one or more diseases after multiple testing correction. Notably, adhesion G protein-coupled receptor G1 (ADGRG1) exhibited a very large effect size (effect size >3.4) in the ARLD and HCC cohorts and a large effect size (effect size ≈2.9) in the VIRAL cohort (Table S8 and Figure 5B). An independent clinical study with a 16-year follow-up reported that ADGRG1 levels were associated with an increased risk of end-stage liver disease.^30^ Additionally, we observed that Fms-related receptor tyrosine kinase 3 (FLT3) was strongly upregulated (effect size ≈4.5) in the AML cohort, while its ligand (FLT3LG) was strongly downregulated (effect size ≈ −3.1; Table S8 and Figure 5B). It is well-established that FLT3 mutations are one of the dominant drivers of AML.^31^

We organized the top proteins in alphabetical order and clustered them based on effect size. The results show that diseases were roughly clustered according to our initial classification into four categories: blood, psychiatric, metabolic, and tumor. This clustering aligns with the expected biological and clinical distinctions among these disease categories (Table S8 and Figure 5B).

### Graph integration and enrichment analysis

**Figure 6:**
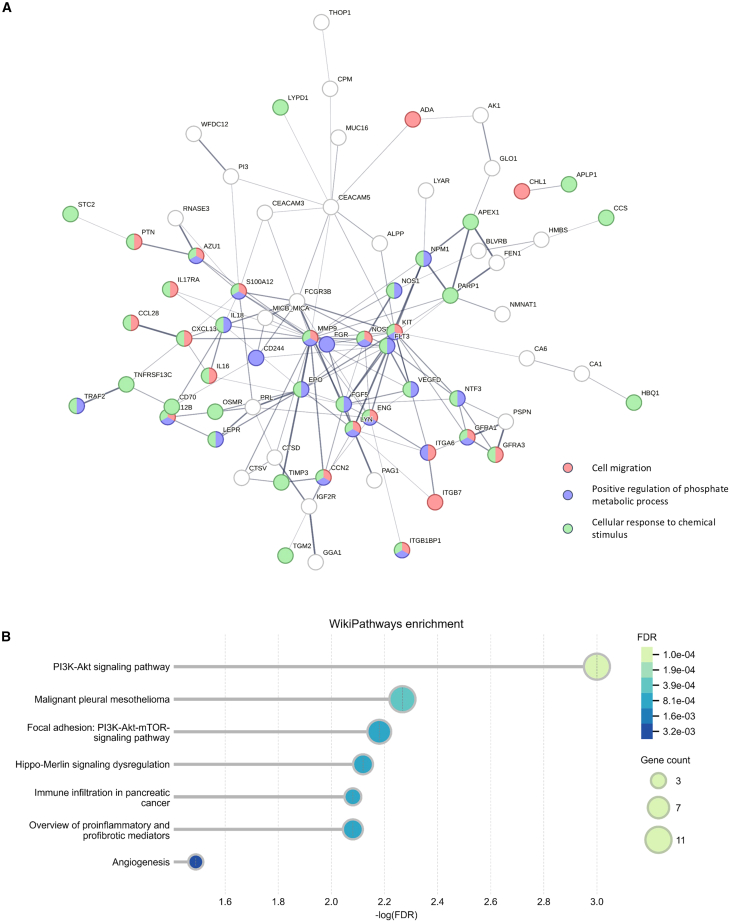
Protein-protein interaction network and pathway enrichment of key proteins (A) The protein-protein interaction (PPI) network formed by the top 100 important proteins from the two-stage hierarchical model, with isolated nodes removed. (B) WikiPathways enrichment analysis for the top 100 proteins from the two-stage hierarchical model.

We constructed a protein-protein interaction (PPI) network for the top 100 proteins identified by our two-stage hierarchical model using the STRING database (Figure 6A). In this network, nodes represent individual proteins, and the thickness of the edges reflects the strength of data support for interactions.^32^ Gene ontology analysis indicated that the majority of these proteins are functionally associated with cell migration, positive regulation of phosphate metabolic processes, and cellular response to chemical stimuli.^33^ Among the identified proteins, some unclassified proteins, such as CEA cell adhesion molecule 5 (CEACAM5), are well-established cancer-related genes. In contrast, prolactin (PRL) has been implicated not only in cancer but also in autoimmune diseases.^34^^,^^35^

To further explore the biological relevance of these proteins, we conducted a WikiPathways enrichment analysis on the top 100 proteins (Figure 6B). The PI3K-Akt signaling pathway emerged as the most significantly enriched, indicated by a high –log(FDR) value and enrichment of 11 associated genes. Notably, seven of these proteins—COL9A1, FGF5, FGFR2, FLT3, FLT3LG, PRL, and TEK—are among the top 40 contributors to disease prediction and were positively associated with more than ten disease groups (Figure 5A). In addition, Hippo merlin signaling dysregulation was also prominently enriched, involving 6 genes. Among these, FGFR2, FLT3, and TEK rank within the top 40 proteins, each contributing positively to predictions across more than ten disease groups (Figure 5A). These pathways are critically involved in cancer and inflammation, regulating key cellular processes such as survival, proliferation, and metabolism, which are often dysregulated in tumorigenesis and inflammatory diseases.^36^^,^^37^^,^^38^ Additionally, other pathways, including malignant pleural mesothelioma, and focal adhesion were also significantly enriched, underscoring their potential relevance to our multi-disease classification.

### Performance evaluation on top-ranked protein subsets

**Figure 7:**
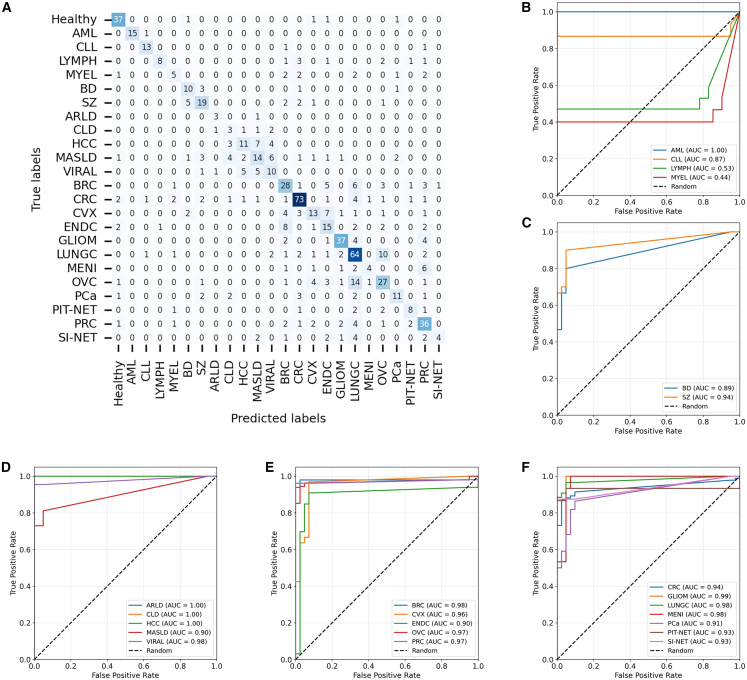
Model performance using the top 100 protein features (A) Confusion matrix of the two-stage hierarchical model evaluated with the top 100 proteins. (B–F) AUROC of the two-stage hierarchical model with the top 100 proteins for binary classification between each disease group and the healthy control group.

After obtaining the protein importance ranking, we retained the model architecture and evaluated the predictive performance of the two-stage hierarchical model when restricted to the top 100 proteins. Under this constraint, the model exhibited a slight but consistent decline in classification performance across all 24 phenotypes (Figures 7A and S2E and Table S9). The overall accuracy reached 0.614, and the weighted F1 score was 0.609—both slightly higher than those of the restricted baseline model (accuracy: 0.606; weighted F1: 0.567). It is worth noting that for a 24-class classification problem, the theoretical accuracy and weighted F1 score of a random guess would be approximately 1/24 (around 0.042), highlighting the robustness of the two-stage model even under feature constraints.

Notably, even with a significantly reduced feature set, the model maintained precision and recall around 0.8 for four disease types: AML, CLL, CRC, and GLIOM (Table S9). We further investigated the performance of the restricted hierarchical model in binary classification settings—where the dataset only included samples from the target disease group and healthy controls. As shown in Figures 7B–7F, with the exception of the LYMPH and MYEL groups, the model continued to demonstrate strong discriminatory power in binary tasks, with AUROC values generally exceeding 0.9. These results suggest that the model holds potential as a clinical decision-support tool for independently identifying disease phenotypes.

### External validation in an independent cohort

To evaluate the generalizability of the hierarchical model beyond the discovery cohorts from Sweden and Turkey, we conducted an external validation using a subset of the UK Biobank. The evaluated phenotypes included CLL, BD, SZ, BRC, CVX, PIT-NET, PRC, and healthy controls.

When applied as a direct multiclass classifier, the model demonstrated very low accuracy in the UK Biobank dataset. Therefore, its performance was further assessed in binary classification settings, analogous to those presented in Figures 3D–3F. The model was able to distinguish patients from healthy controls with reasonable performance (AUC >0.7; Figure S3A) for five diseases—CLL, BRC, CVX, PIT-NET, and PRC. In contrast, no discriminative ability was observed for BD and SZ (AUC = 0.5; Figure S3B).

The suboptimal performance in this external cohort is likely attributable to two main factors. First, domain drift between proteomic measurement platforms: Olink PEA quantifies proteins in NPX units rather than absolute concentrations, potentially introducing systematic shifts that are challenging to correct without domain adaptation strategies. Second, limited training sample sizes for certain phenotypes, particularly BD and SZ, may have hindered the model’s ability to learn robust molecular discriminants.

### Assessment of potential confounding by demographic and clinical variables

To evaluate whether demographic variables influenced model performance, we performed post hoc stratification analyses for age, sex, and BMI. Information on comorbidities was not available in the original dataset, and therefore, could not be directly adjusted for.

First, to avoid trivial bias from sex-specific diseases, phenotypes inherently associated with biological sex (BRC, CVX, ENDC, PRC, and OVC) were excluded, leaving 19 phenotypes for comparison. Test samples were split into male-only and female-only subsets, and model performance was assessed separately. Performance metrics (precision, recall, and F1 score) were computed using classification_report from sklearn.metrics. Group-level comparisons were performed with the Mann-Whitney U test (α = 0.01). No significant differences were observed between sexes: precision *p* = 0.94, recall *p* = 0.71, F1 score *p* = 0.58.

Second, individuals were stratified into older (≥60 years) and younger (<60 years) subgroups. Performance metrics were compared between these age strata using the Mann-Whitney U test (α = 0.01). Differences were not statistically significant: precision *p* = 0.74, recall *p* = 0.49, F1 score *p* = 0.49.

Lastly, participants were stratified by BMI into BMI >25 and BMI ≤25 groups. Comparisons of precision, recall, and F1 score again showed no significant differences (Mann-Whitney U test, α = 0.01): precision *p* = 0.84, recall *p* = 0.53, F1 score *p* = 0.72.

We note that several of the top 40 proteins shown in Figure 5 (for example, GFAP, TNFRSF11B, CGA) exhibit significant Spearman correlations with clinical variables (Figure S3C). However, the relationships between NPX values and clinical variables were nonlinear, and substantial NPX variance remained within clinical strata (Figures S3D–S3F). Thus, although demographic variables correlate with some proteomic features, they do not fully account for the proteomic signals exploited by the model.

### Sensitivity analysis

To evaluate the stability of the hierarchical model—specifically, whether its characterization of healthy samples depends on a small subset of individuals—we performed a sensitivity analysis based on repeated random subsampling.

Among the 96 healthy samples in the training set (70% split), we randomly removed one-quarter (24 samples) in each iteration and retrained the hierarchical model. This random deletion and retraining procedure was repeated 50 times, and performance metrics were recorded (Table S11). As expected, both accuracy and weighted F1 score showed small decreases across iterations (0.007–0.02), likely reflecting the reduction in training sample size and the absence of hyperparameter retuning for each re-trained model.

Interestingly, despite the reduction in healthy samples, the model’s ability to correctly identify healthy individuals did not decrease. In some iterations, the F1 score for the healthy class even reached 1. In contrast, the slight drop in overall performance arose primarily from weaker classification of disease samples. This pattern suggests that the removed healthy samples may have been entangled with certain disease samples in the high-dimensional proteomic space.

To further evaluate this hypothesis, we applied the DeLong test, whose null hypothesis states that the difference between the areas under two correlated ROC curves is zero. Across the 50 subsampling iterations, depending on the disease phenotype, approximately one-quarter to one-half of the subsampled models showed significant AUC changes for the healthy-disease subcohorts. This indicates that some of the removed healthy samples carried discriminative proteomic patterns that were not uniformly present across all healthy individuals.

## Discussion

Leveraging insights from pathology, we developed a knowledge-based hierarchical classifier using proteomics data. Our results demonstrate that this hierarchical classifier effectively distinguishes 23 common diseases, including blood diseases, psychiatric disorders, metabolic diseases, and tumors. Notably, the hierarchical classifier outperforms a single explicit multi-class machine learning model in almost all specific disease classifications. Integrating pathological knowledge with machine learning models demonstrates significant advantages over using proteomics data alone. Based on feature importance analysis, certain proteins, such as FCGR3B, exhibit substantial discriminative value across various diseases (Figure 5A), even though they do not show statistically significant changes compared to the healthy cohort (Figure 5B). This suggests that machine learning models may uncover information that traditional statistical hypothesis testing cannot capture. These improvements in discrimination could translate into clinical utility, offering potential benefits for a wide range of decision-making processes.

As evident from Figure 5B, it is unlikely that a single plasma protein can simultaneously predict the risk of multiple disease events, whether from a statistical or machine learning perspective. We overcame the limitations of proteomics technology by constructing a two-stage hierarchical model based on 1,462 proteins, which outperformed traditional machine learning models in classifying disease phenotypes. Traditional statistical machine learning models often rely on controlling certain variables, such as age and gender. While this is ideal, such information is frequently partially missing in large-scale, multi-center studies due to various reasons. The input for our two-stage hierarchical model is solely proteomics data, making it independent of individual metadata and thus more scalable. Furthermore, compared to traditional models, our knowledge-based model actively directs attention to distinctions within similar phenotypes rather than simply minimizing the loss function in a general manner. This approach ensures that the identified set of important features does not favor or neglect specific disease types.

Previous studies have demonstrated the high predictive value of proteomics analysis for various health conditions, including blood diseases,^39^^,^^40^ psychiatric disorders,^41^^,^^42^ metabolic diseases,^11^^,^^43^^,^^44^ and tumors.^45^^,^^46^^,^^47^ However, earlier research has often focused on specific diseases rather than a broader pan-disease perspective. Álvez et al. extended this effort to a pan-cancer proteomics model using a linear model.^4^ Here, we take a further step by considering a wider range of conditions, including cancer, inflammatory diseases, and poorly defined psychiatric disorders. To our knowledge, this is the first study to broadly reveal the predictive power of blood proteomics for multi-disease outcomes by integrating fundamental pathological knowledge. Importantly, we found that for nearly all the diseases we examined, the knowledge-based hierarchical model demonstrated predictive performance comparable to or slightly better than traditional models, particularly for disease phenotypes with small sample sizes. Furthermore, as shown in Figures 3D–3F, S2B, and S2C, the hierarchical model alone achieved near-perfect classification for most diseases compared to healthy controls, although it exhibited limitations in multi-class classification, similar to classical models. Our findings strongly emphasize the predictive value of blood proteomics as a single-source, personalized health screening tool, reducing the need for multi-dimensional data collection required for separate disease testing. Our hierarchical model directly enhances interpretability by mirroring clinical diagnostic workflows—first categorizing diseases into clinically meaningful groups, then refining subtypes within each group—while SHAP-based protein importance scores identify biologically plausible decision drivers. Therefore, proteomics analysis holds significant promise as a replacement for complex laboratory tests or clinical evaluations, potentially improving risk assessment for multiple diseases simultaneously.

Plasma biomarkers reflecting human health status are central to clinical decision-making. Conventional biomarkers are often specific to particular diseases and are typically compared against healthy samples. However, Welch’s *t* test reveals that large sample variances can lead to test statistics approaching zero, resulting in nonsignificant hypothesis testing outcomes.^48^ Since permutation importance does not rely on pre-assumed significance levels, it allows us to derive results that differ from classical statistical approaches. We found that ITGB1BP1 plays a significant predictive role across multiple disease categories, a finding supported by other studies (Figure 5A). Recent research has shown that ITGB1BP1 is a novel transcriptional target of CD44-downstream signaling, promoting cancer cell invasion.^49^ Earlier studies have also demonstrated that PAK proteins and YAP-1 signaling downstream of integrin beta-1 in myofibroblasts promote liver fibrosis.^27^ Similarly, we observed that FCGR3B (also known as CD16 or CD16b) is highly important in model predictions, contributing positively to the prediction of 18 diseases (Figure 5A). FCGR3B is a marker of natural killer cells and activated macrophages/monocytes. Increased macrophages and altered brain endothelial cell gene expression have been reported in the frontal cortex of individuals with schizophrenia displaying inflammation.^50^ Additionally, copy number variation of FCGR3B and FcγR polymorphisms are associated with susceptibility to systemic autoimmunity.^26^^,^^51^ Our study provides preliminary evidence for the predictive value of these proteins across multiple diseases, and future research is needed to validate our observations.

Our hierarchical model also outperforms the baseline model in interpreting multi-protein interactions. While the baseline approach identifies the top 100 most contributive proteins and broadly associates them with proinflammatory and profibrotic mediators—a connection that is reasonable but overly generalized—our hierarchical model reveals a more specific and biologically coherent subset: FGFR2, FLT3, KIT, and TEK (with KIT ranked among the top 100 but not within the top 40 contributors). Notably, all four proteins are members of the receptor tyrosine kinase (RTK) family. RTKs constitute a major class of cell surface receptors that regulate essential cellular processes such as proliferation, survival, and differentiation through activation of downstream pathways including PI3K/AKT and MAPK.^52^^,^^53^ Dysregulation of RTK signaling—through gene amplification, mutation, or ligand overexpression—is a well-established driver of oncogenesis across multiple tumor types.^53^^,^^54^^,^^55^ NF2, which encodes the tumor suppressor Merlin, acts as a critical negative regulator of RTK-mediated signaling. In addition to modulating RTK outputs, Merlin functions as an upstream activator of the Hippo pathway, which limits cell growth by inhibiting the transcriptional co-activators YAP/TAZ.^56^ Loss of NF2 results in hyperactivation of both RTK-dependent and Hippo-independent proliferative signaling, thereby contributing to tumor initiation and progression.^57^ Recently, RTK dysregulation has also been implicated in non-neoplastic diseases: alterations in FGFR, EGFR, and MET pathways have been linked to neurodevelopmental and psychiatric disorders such as schizophrenia, depression, and autism spectrum disorder.^58^ Furthermore, RTKs including MET and FGFR4 play key roles in hepatic metabolism and regeneration, with aberrant signaling contributing to non-alcoholic fatty liver disease and liver fibrosis.^59^ These findings highlight the functional interplay between RTK signaling, Hippo pathway regulation, and NF2 status, not only in tumor biology but also in broader disease contexts, and support further exploration of combinatorial therapeutic strategies in both NF2-deficient cancers and RTK-associated metabolic or neuropsychiatric disorders.

### Limitations of the study

This study has several strengths, including the application of high-throughput proteomics technology, a large multi-disease cohort, and a comprehensive evaluation of multi-center independent sampling. However, there are also some limitations worth discussing. First, some proteins not included in the Olink panels but potentially predictive of multiple disease outcomes may have been overlooked. Nevertheless, the aim of this study was to assess the feasibility of machine learning classification based on proteomics data in clinical applications rather than to discover new proteins. Second, the majority of participants in this study were of Northern European and Turkish descent. Although we performed internal cross-validation and all models were well-calibrated, similar to research in biomedicine and other applied fields, we emphasize the need for future analyses using large-scale, synchronized regional datasets to develop universally applicable models, despite the strong predictive performance of the proposed knowledge-based hierarchical model.^18^^,^^60^ Third, although our downsampling analysis suggested that the high binary classification performance (AUROC >0.9) was robust to the limited healthy control sample size (*n* = 137), future studies with larger population-based control cohorts are warranted to confirm these findings. Caution should be exercised when generalizing the model to screening applications where disease prevalence is low. Finally, although we examined potential demographic influences through post hoc stratification by age, sex, and BMI, our dataset did not include complete information on comorbidities or other clinical covariates. Consequently, we were unable to perform explicit confounder adjustment within the modeling framework. Future studies incorporating harmonized demographic and comorbidity data will be essential for explicitly adjusting for potential confounders and for further validating the generalizability of our hierarchical model across broader and more diverse populations.

Despite these limitations, blood proteomics has shown significant advantages in multi-disease modeling, exhibiting ideal predictive performance for a variety of diseases. These discriminative capabilities can largely translate into practical clinical utility. In summary, our work highlights the critical potential of plasma proteomics data in statistical machine learning analysis, enabling the simultaneous identification of multiple diseases.

## Resource availability

### Lead contact

Requests for further information and resources should be directed to and will be fulfilled by the lead contact, Adil Mardinoglu (adilm@kth.se).

### Materials availability

This study did not generate new materials or unique reagents.

### Data and code availability

The dataset analyzed in this study is a subset of the pan-disease proteomic atlas generated by M.U. and colleagues. Individual-level raw clinical and proteomic data cannot be shared publicly due to data protection regulations. Aggregated summary data are available through the Human Disease Blood Atlas (www.proteinatlas.org). Access to the full dataset for validation purposes can be requested, subject to appropriate ethical approval and data-use agreements: https://doi.org/10.17044/scilifelab.28577390.v1.

The machine learning results were generated using Python version 3.11.11 with scikit-learn (sklearn) version 1.4.2. Detailed information about the library versions and computational environment can be found in the GitHub repository (https://github.com/lingqime/protein_hierarchical_model).

Any additional information required to reanalyze the data reported in this article is available from the lead contact upon request.

## Acknowledgments

This work was supported by the 10.13039/501100004063Knut and Alice Wallenberg Foundation (grant nos. 72110 to A.M. and KAW2022.0318 to M.U.).

## Author contributions

Conceptualization, A.M.; data curation, M.B.A., O.A., and H.T.; formal analysis, L.M. and M.L.; funding acquisition, A.M. and M.U.; investigation, M.B.A., O.A., and H.T.; methodology and supervision, C.Z. and A.M.; validation, X.K., T.Z., X.L., C.Z., and A.M.; visualization, L.M. and X.K.; writing – original draft, L.M., M.L., and A.M.; writing – review and editing, all authors.

## Declaration of interests

The authors declare no competing interests.

## Declaration of generative AI and AI-assisted technologies in the writing process

During the preparation of this work the authors used ChatGPT in order to improve readability and language. After using this tool/service, the authors reviewed and edited the content as needed and take full responsibility for the content of the published article.

## STAR★Methods

### Key resources table

**Table:** 

REAGENT or RESOURCE	SOURCE	IDENTIFIER
**Software and algorithms**		

Two-stage hierarchical machine learning model	This paper	https://github.com/lingqime/protein_hierarchical_model
Python 3.11.11	Python Software Foundation	https://www.python.org/downloads/release/python-31111/
scikit-learn 1.4.2	Python Software Foundation	https://pypi.org/project/scikit-learn/1.4.2/

**Other**		

The training and testing cohort	Human Disease Blood Atlas	https://v22.proteinatlas.org/humanproteome/disease
UK biobank data set	UK Biobank	https://www.ukbiobank.ac.uk/

### Experimental model and study participant details

The dataset analyzed in this study is a subset of the pan-disease proteomic atlas generated by Prof. Mathias Uhlén and colleagues. Individual-level raw clinical and proteomic data cannot be shared publicly due to data protection regulations. The clinical information for each phenotype can be seen in Table S1. Aggregated summary data are available through the Human Disease Blood Atlas (www.proteinatlas.org). Access to the full dataset for validation purposes can be requested, subject to appropriate ethical approval and data-use agreements: https://doi.org/10.17044/scilifelab.28577390.v1.

To assess the potential influence of clinical variables on study outcomes, post hoc stratification analyses were performed based on sex, age, and BMI. No significant differences in model performance were observed across these strata, indicating that the results were not systematically associated with sex, age, or BMI.

The machine learning results were generated using Python version 3.11.11 with scikit-learn (sklearn) version 1.4.2. Detailed information about the library versions and computational environment can be found in the GitHub repository (https://github.com/lingqime/protein_hierarchical_model).

### Method details

The research complies with all relevant ethical regulations. The pan-cancer study was approved by the Swedish Ethical Review Authority (EPM dnr 2019-00222). The research was in line with donor consents in U-CAN (28631533, EPN Uppsala 2010198 with amendments), and all participants provided written informed consent. The study protocol conforms to the ethical guidelines of the 1975 Declaration of Helsinki.

#### The pan-cancer study cohort

Plasma samples from 1477 cancer patients were obtained from the U-CAN biobank which collects samples from consenting patients diagnosed at the Akademiska hospital in Uppsala as part of the clinical routine and with a high degree of standardization. Plasma samples were obtained from treatment-naïve patients taken around the time of their diagnosis. Plasma was prepared from whole blood by centrifugation at 2.400 × g for seven minutes at room temperature, after which the plasma was aliquoted into several 220 μl vials and immediately frozen for long-term storage at −80 °C. Exclusion criteria included any concurrent or previous cancer within the last five years, and arm-to-freezer time exceeding 360 min. Diagnosis, stage, age, sex and other variables were obtained from the U-CAN database and the patient’s clinical records.

#### Measurement of protein levels

The protein levels of all 3,879 samples were measured in plasma using the Olink Explore PEA technology. This technology leverages antibody-binding capabilities to detect the levels of 1,462 protein targets in plasma, coupled with next-generation sequencing (NGS) readout. The Olink Explore 1536 platform comprises four distinct panels: the Olink Explore 384 Cardiometabolic Reagent Kit, the Olink Explore 384 Inflammation Reagent Kit, the Olink Explore 384 Oncology Reagent Kit, and the Olink Explore 384 Neurology Reagent Kit. A total of 1,472 proteins were targeted using specific antibodies, including 1,462 unique proteins and additional controls. Each antibody was conjugated separately with two complementary probes and distributed across the four 384-plex panels, each focusing on one of the four disease areas: cardiovascular, inflammation, neurology, and oncology.

The PEA workflow began with an overnight incubation to allow the conjugated antibodies to bind to their corresponding proteins in the samples. This was followed by an extension and pre-amplification step, during which hybridization and extension of complementary probes occurred. The extended DNA was then amplified by PCR and indexed to prepare sequencing libraries, which were subsequently sequenced using Illumina’s NovaSeq platform. The raw counts obtained from sequencing underwent a rigorous quality control and normalization process. Internal controls were introduced at various steps to minimize intra-assay variability. These included (i) an incubation control – consisting of a non-human antigen measured using the same technology; (ii) an extension control—comprising an antibody conjugated to a unique pair of probes designed to produce a positive signal upon proximity; and (iii) an amplification control – consisting of a double-stranded DNA sequence expected to produce a positive signal independently of the amplification step.

Additionally, external controls, such as negative controls (buffer samples) and plate controls (pooled plasma samples), were used to establish the limit of detection (LOD) and adjust protein levels between plates, respectively. Two known samples served as sample controls to assess measurement precision. Following quality control and normalization, the data were reported in Normalized Protein eXpression (NPX) units, a relative protein quantification metric on a log2 scale. The NPX score was derived from matched sequencing counts, with higher NPX values indicating higher protein levels. Measurements that failed internal quality control checks and were flagged with warnings were excluded from the dataset.

For quality assurance, three protein assays (IL6, CXCL8, and TNF) were included in all four panels. These served as technical controls, enabling the investigation of sample quality through interpanel correlation analysis for NPX values above the LOD. Furthermore, the coefficient of variation (CV) for each assay was calculated to quantify technical variance within a plate (IntraCV) and across multiple plates (InterCV). This calculation was based on pooled plasma samples run in duplicate on each plate, following the methodology described in the Wik et al.^63^

#### Algorithm description in mathematics

Let ${\left\{\left(x_{i},y_{i}\right)\right\}}_{i=1}^{N}$ be our dataset, where $x_{i}\in R^{n}$ and *y*_*i*_∈{1,…,*M*}. We first partition the set {1,…,*M*} into *K* disjoint categories, such that they form equivalence classes. Let ${\bar{y}}_{i}$ denote the equivalence class of *y*_*i*_; then the set ${\left\{{\bar{y}}_{i}\right\}}_{i=1}^{N}$ contains exactly *K* distinct elements. We train the first-layer model on the dataset ${\left\{\left(x_{i},y_{i}\right)\right\}}_{i=1}^{N}$, and the second-layer model on the dataset ${\left\{\left(X_{j},y_{j}\right)\right\}}_{j=1}^{K}$, where $X_{j}=\left\{x|\left(x,y\right)\;\text{is}\;a\;\text{paired}\;\text{data}\;\text{point}\;\text{with}\;y\in {\bar{y}}_{j}\right\}$. Let *f* be the well-trained function on the first layer, and let *g*_*j*_ be the well-trained functions on the second layer. The final prediction of the two-layer hierarchical model is given by

$$P\left(X\right)=\sum_{j=1}^{K}P_{f}\left(X\right)\cdot P_{g_{j}}\left(X_{j}|f\left(X\right)\right)\text{,}$$

where $P_{f}\left(X\right)$ is the probability output of the first-layer model, and $P_{g_{j}}\left(X_{j}|f\left(X\right)\right)$ is the conditional probability output of the second-layer model given the output of the first layer.

In particular, when the first-layer model yields high accuracy, i.e., $P\left(f\left(x_{i}\right)=y_{i}\right)\approx 1$, we have

$$P\left(X\right)=P_{f}\left(X\right)\cdot P_{gf\left(X\right)}\left(X_{f\left(X\right)}|f\left(X\right)\right)\text{.}$$

Throughout the paper, we assume that the function families of interest belong to the collection F, which corresponds to the models in the scikit-learn library. That is, $f,g_{j}\in F$.

During the training process, we addressed the issue of imbalanced samples by applying the Borderline-SMOTE algorithm, implemented using the Python library imblearn. The detailed methodology is outlined in the Han et al.,^24^ and we provide a brief description of the algorithm here. Let the minority class samples be denoted as $S_{\min}=\left\{x_{n_{1}},x_{n_{2}},\ldots ,x_{n_{l}}\right\}$. For each minority sample $x_{n_{i}}\in S_{\min}$, the following steps are performed: (i) Compute the *k*-nearest neighbors *N*_*k*_(***x***_*i*_) using the Euclidean distance metric; (ii) Count the number of majority class samples in *N*_*k*_(***x***_*i*_), denoted as *m*_*i*_. For each minority sample $x_{n_{i}}$ satisfying the condition $\frac{k}{2}\leq m_{i}<k$, a minority sample ***x***_*z*_ is randomly selected from its *k*-nearest neighbors. A synthetic sample ***x***_new_ is then generated as follows

$$x_{\text{new}}=x_{n_{i}}+\lambda \cdot \left(x_{z}-x_{n_{i}}\right)\text{,}$$

where *λ*∈[0,1] is a randomly generated number. This process is repeated until the minority class reaches the desired size, resulting in a balanced dataset.

#### Permutation feature importance

We employed permutation feature importance to evaluate the significance of individual features, which is a special case of perturbation.^61^^,^^62^ Specifically, let *F* denote the final well-trained model, and let the test dataset be represented as

$$X_{\text{test}}=\left\{x_{n_{1}},x_{n_{2}},\ldots ,x_{n_{k}}\right\}\text{.}$$

We first compute the score of the model on the test data, *s*(*F*(*X*_test_),*y*_test_). To assess the importance of the *i*-th feature, we randomly permute the elements of the *i*-th row of *X*_test_ and repeat this process *P* times. Theoretically, the importance score for the *i*-th feature is given by:

$$\frac{1}{n!}\sum_{\sigma_{i}}\left(s\left(F\;\left(X_{\text{test}}\right),y_{\text{test}}\right)-s\left(F\;\left(\sigma_{i}\left(X_{\text{test}}\right)\right),y_{\text{test}}\right)\right)$$

where *σ*_*i*_ represents a permutation of the *i*-th row. However, since *n*= 1462 (making *n*! computationally infeasible), we approximate this by performing 1000 random permutations instead.

#### Two-stage hierarchical classification model

In the first stage, a Perceptron was employed as the foundation framework. In the second stage, different classification algorithms were selected based on disease categories: for blood diseases, the Extra Trees Classifier was used; for psychiatric diseases, the Stochastic Gradient Descent (SGD) Classifier was adopted; for metabolic diseases, the Ridge Classifier was applied; and for cancers, the Logistic Regression model was utilized. We chose these models from the process described below.

Our candidate models included 20 commonly used machine learning classifiers (based on our limited experience and understanding, with no intention of excluding other valid models): AdaBoostClassifier, BaggingClassifier, BernoulliNB, DecisionTreeClassifier, DummyClassifier, ExtraTreesClassifier, GaussianNB, KNeighborsClassifier, LabelPropagation, LGBMClassifier, LinearDiscriminantAnalysis, LogisticRegression, MLPClassifier, NuSVC, Perceptron, RandomForestClassifier, RidgeClassifier, SGDClassifier, SVC, and XGBClassifier. All models were implemented using Python’s scikit-learn library.

To identify the most suitable models for each stage and disease category, we split the original dataset into a 70% training-validation set and a 30% independent test set. Within the training-validation set, we performed 5-fold cross-validation to evaluate each model. Specifically, the data were divided into five equal parts; in each iteration, four parts (56%) were used for training and one part (14%) for validation, and this process was repeated five times. The mean F1 scores from the five folds were then computed and used to rank the 20 candidate models in descending order (Table S10). Regarding the model training strategy, the two-stage hierarchical model was trained in separate rounds rather than simultaneously. This design choice reflects the fact that different machine-learning algorithms have distinct loss functions, and there is no unified loss function that would allow joint optimization of all sub-models simultaneously.

During training, we considered both the raw training-validation set and oversampled versions using various oversampling algorithms (Borderline-SMOTE, ADASYN, and SMOTEENN). We observed that for overall phenotype classification (where LogisticRegression performed best), metabolic diseases (RidgeClassifier performed best), and cancers (LogisticRegression performed best), the use of oversampling did not affect model ranking. However, for blood and psychiatric diseases, the choice of oversampling technique significantly influenced model performance rankings (Table S10).

To ensure methodological consistency, we applied the same oversampling method (Borderline-SMOTE) across all cases. Under this unified approach, SGDClassifier became the top-performing model for psychiatric diseases. Based on similar reasoning, we selected Perceptron for the first-stage classifier, RidgeClassifier for metabolic diseases, and LogisticRegression for cancers. In total, four different classifiers were employed across categories.

For blood diseases, although RidgeClassifier ranked first and LogisticRegression second, we chose ExtraTreesClassifier (third place, only 0.01 lower in F1 score) to maintain a consistent and aesthetically coherent model ensemble. We considered this minor difference negligible given potential stochastic variation in data, and we expected the nonlinear nature of ExtraTreesClassifier to offer additional modeling flexibility (Table S10).

We further tested how replacing ExtraTreesClassifier with RidgeClassifier would affect the hierarchical model’s overall performance. As expected, after performing 5-fold cross-validation with RidgeClassifier, the overall accuracy remained unchanged, the macro-F1 score increased by 0.0003, and the weighted-F1 score decreased by 0.00005 (Table S10). This change was solely due to one sample (a patient with colorectal cancer) being misclassified as CLL instead of healthy control. Therefore, the overall accuracy did not change, and the variation in macro and weighted F1 scores was negligible.

### Quantification and statistical analysis

The false discovery rate (FDR) was corrected using the Benjamini–Hochberg procedure, and the results were evaluated using StringDB. In Figure 5, we assumed that the data for each protein followed a normal distribution, allowing us to apply the t-test. The resulting p-values were adjusted using the Bonferroni correction, where the corrected p-value is calculated as *p*_*corrected*_=*p*× 1462.

### Additional resources

The machine learning results were generated using Python version 3.11.11 with scikit-learn (sklearn) version 1.4.2. Detailed information about the library versions and computational environment can be found in the GitHub repository (https://github.com/lingqime/protein_hierarchical_model).

## Supplemental information

Document S1. Figures S1–S3

Table S1. Clinical attributes of the cohort, related to Figures 2 and S1

Table S2. Hierarchical model performance on the testing cohort, related to Figure 3

Table S3. Confusion matrix of hierarchical model on the test cohort, related to Figure 3

Table S4. Baseline model performance on the test cohort without Borderline-SMOTE, related to Figure 4

Table S5. Baseline model performance on the test cohort with Borderline-SMOTE, related to Algorithm comparison

Table S6. Top feature importance of baseline model, related to Figure 4

Table S7. Feature importance of hierarchical model for various diseases, related to Figure 5

Table S8. Effect size between the disease cohort and healthy cohort for the top 40 proteins, related to Figure 5

Table S9. Hierarchical model performance with restricted feauture number, related to Figure 2

Table S10. Model selection, related to STAR Methods

Table S11. Sensitivity analysis and Mann-Whitney U test, randomly remove 24 healthy samples each time, related to Sensitivity analysis
