# Supplementary material for: Machine learning identifies proteomic risk factors across 23 diseases
Source: iScience. 2026 Jan 14;29(2):114687. doi: 10.1016/j.isci.2026.114687 (PMC12874149; doi:10.1016/j.isci.2026.114687)
Supplement: Document S1. Figures S1–S3 [file mmc1.pdf]

iScience, Volume *29*

Supplemental information

Machine learning identifies proteomic risk factors across 23 diseases

Lingqi Meng, Mengzhen Li, Xiangtai Kong, Tonghua Zhang, María Bueno Álvez, Xinmeng Liao, Hasan Türkez, Ozlem Altay, Cheng Zhang, Mathias Uhlén, and Adil Mardinoglu

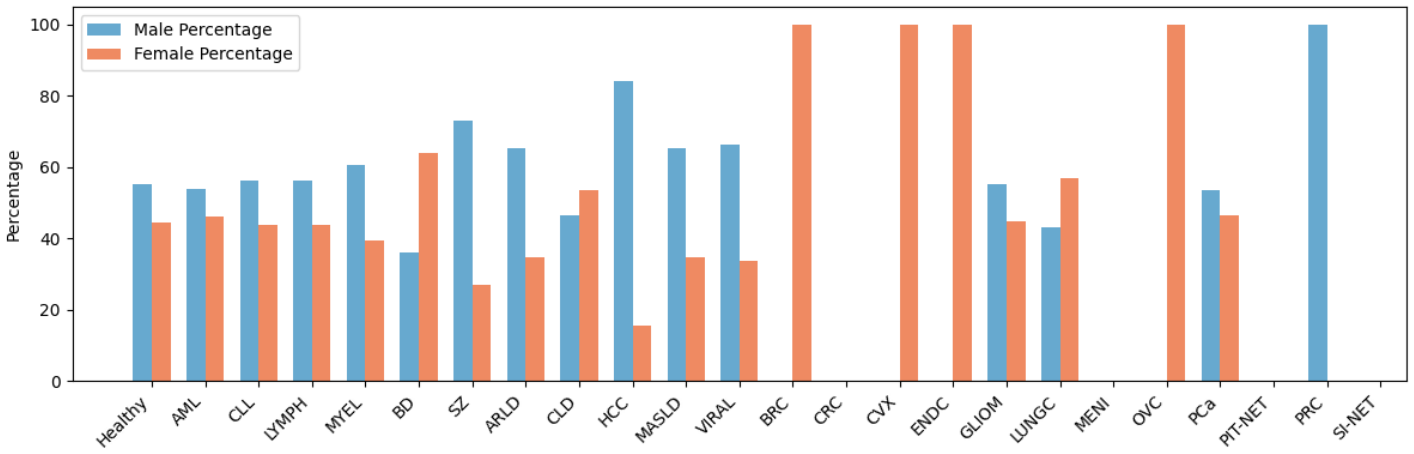

Figure S1. Sex distribution across each disease.

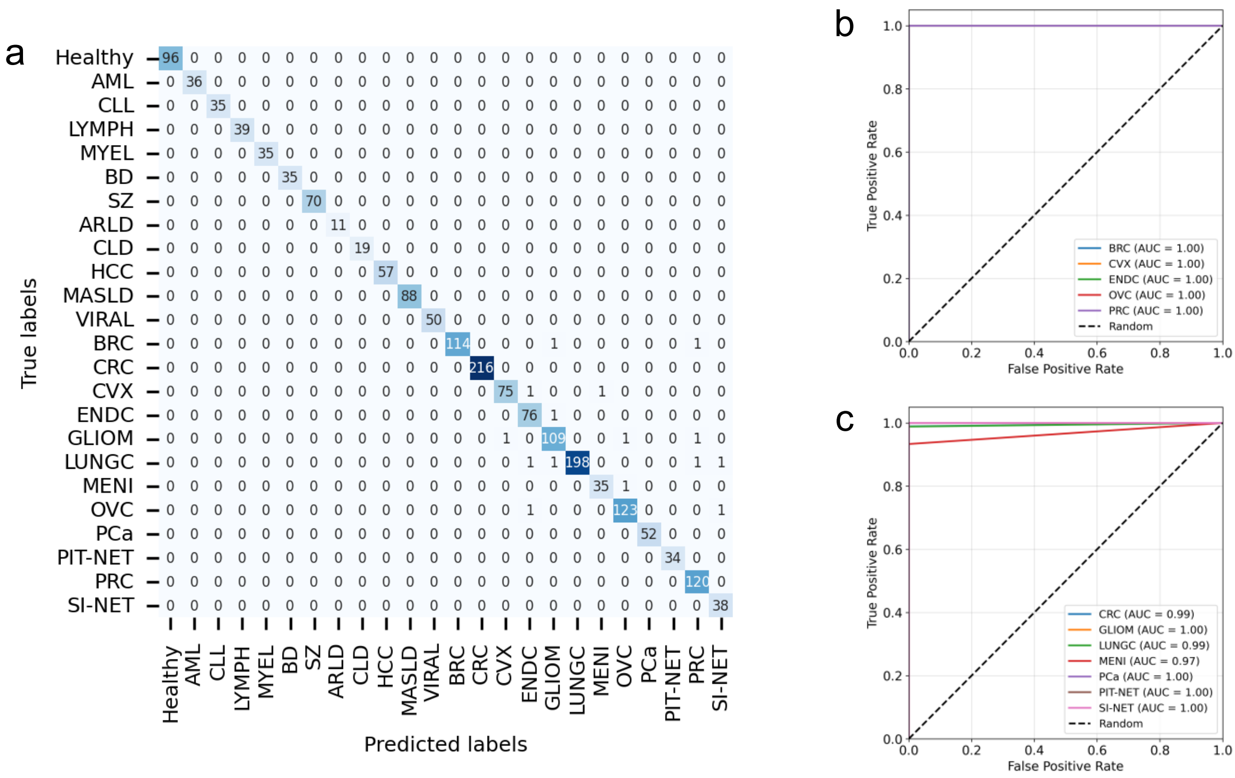

Figure S2. Performance evaluation of the two-stage hierarchical classification model. (a) Confusion matrix of the two-stage hierarchical model on the training cohort. (b-c) AUROC of the two-stage hierarchical model restricted to binary classification of tumor versus healthy cohorts. (d) Classification performance of logistic regression with borderline-SMOTE. (e) Classification performance of hierarchical model on top-ranked protein subsets with borderline-SMOTE.

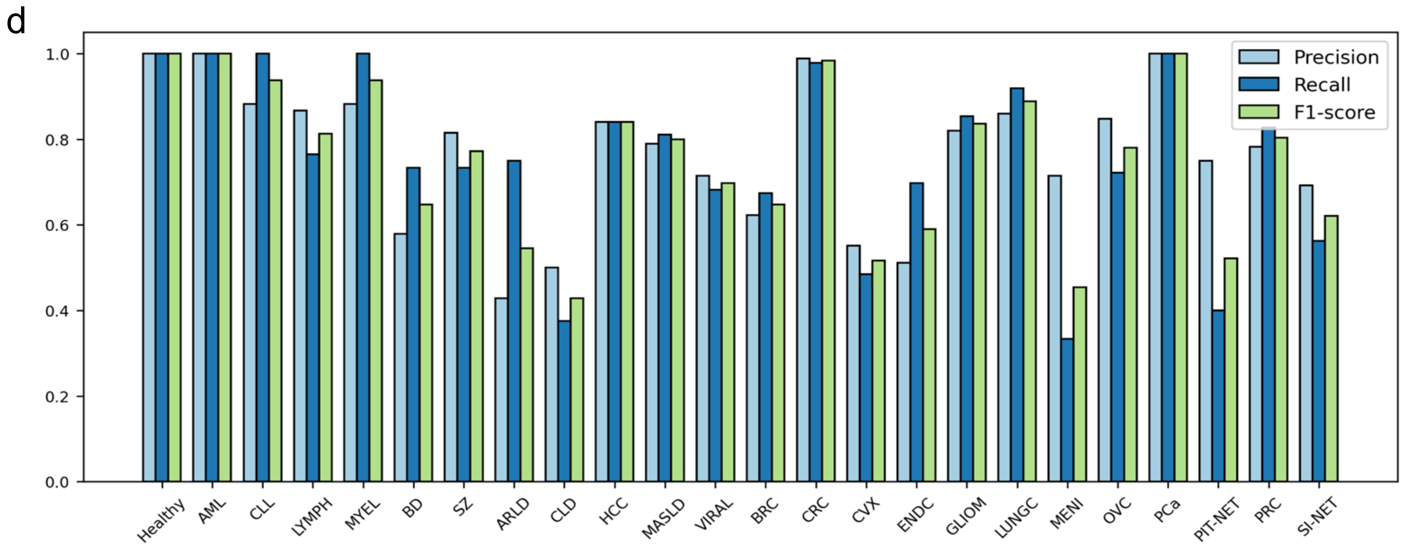

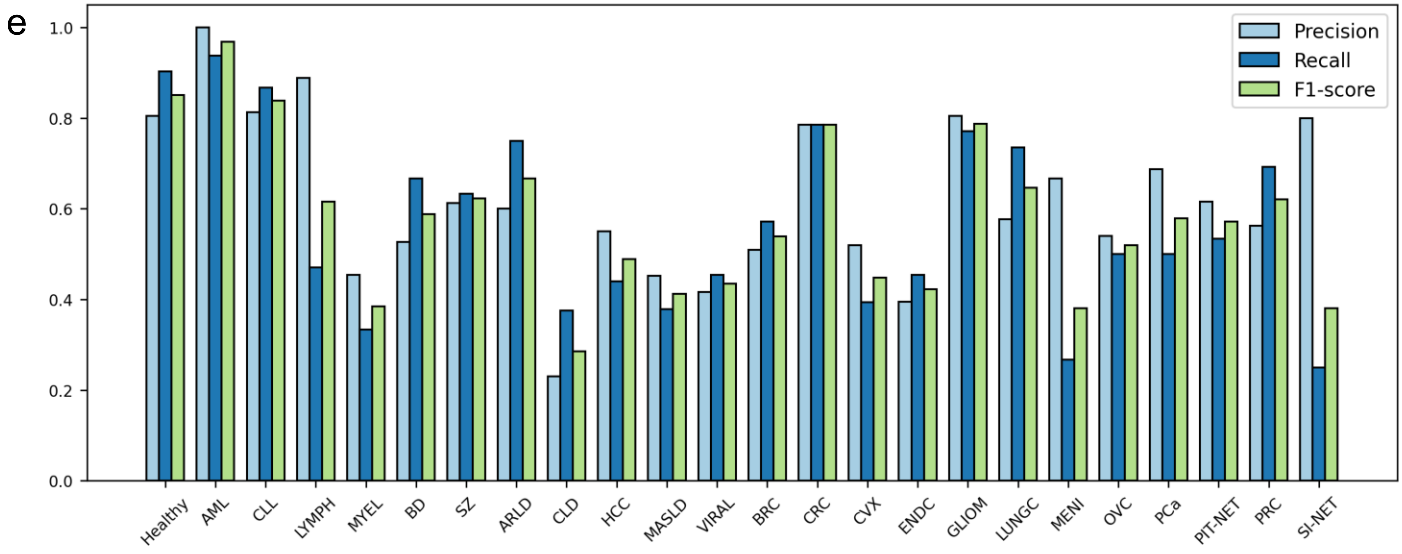

Figure S2. (continued)

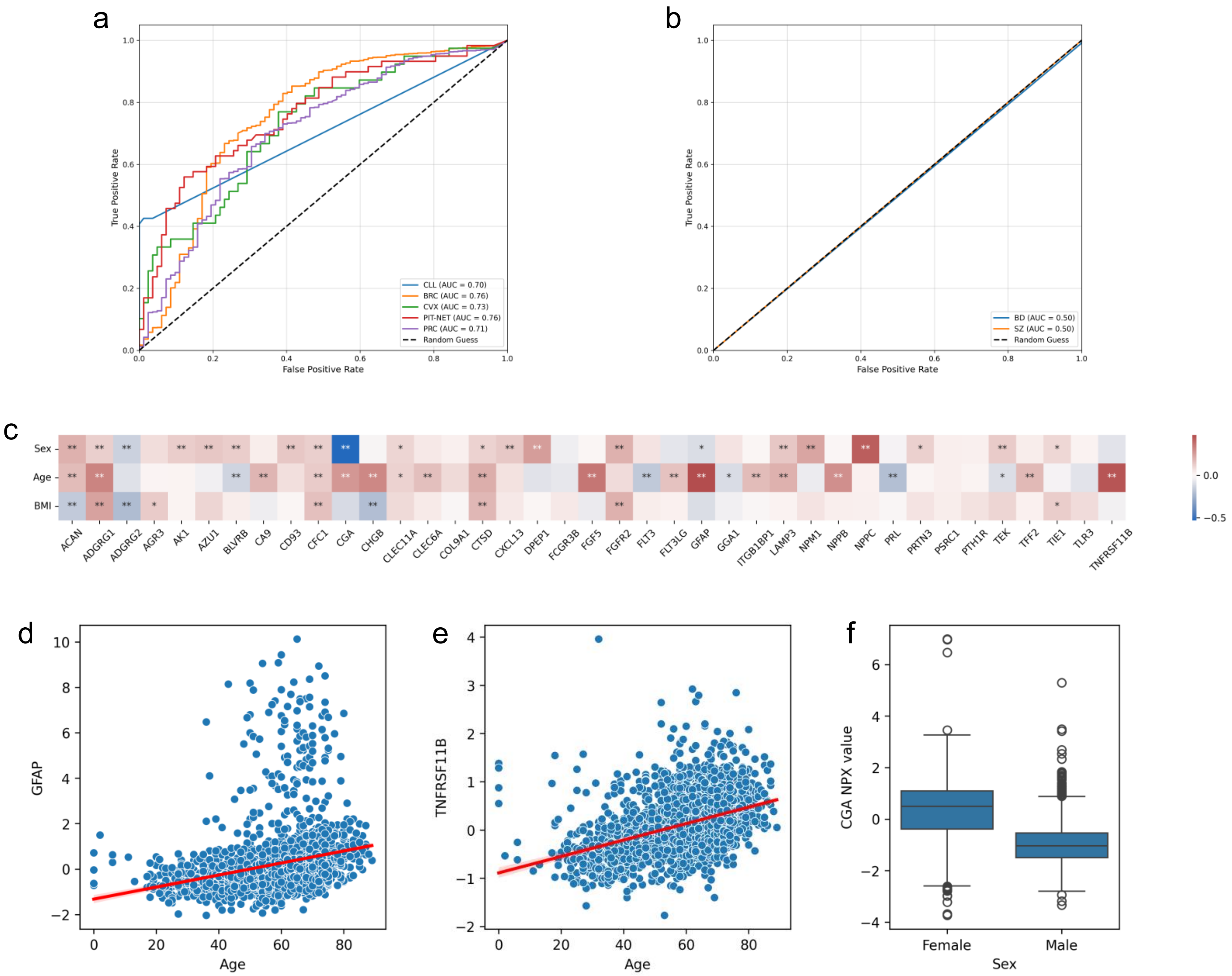

Figure S3. External validation on UK Biobank dataset and demographic association analyses. (a-b) AUROC of the two-stage hierarchical model for binary classification of specific diseases versus healthy controls in the UK Biobank dataset: (a) tumors and (b) psychiatric disorders. (c) Spearman correlations between clinical variables (sex, age, and BMI) and the top 40 proteins. (d–e) Regression plots showing the association of age with (d) GFAP and (e) TNFRSF11B. (f) Box plot comparing CGA levels by sex.
